# Cognitive control training as an add-on treatment for late-life depression: A multicenter randomized controlled trial

**DOI:** 10.1016/j.invent.2026.100951

**Published:** 2026-05-16

**Authors:** Yannick Vander Zwalmen, Janna N. Vrijsen, Bart Meuleman, Marie-Anne Vanderhasselt, Paul Naarding, Rose M. Collard, Marleen van Beek, Peter Oostelbos, Linda Bolier, Dorine van Driel, Jan Spijker, Eni S. Becker

**Affiliations:** a Department of Experimental Clinical and Health Psychology, Ghent University, Ghent, Belgium; b Behavioural Science Institute, Radboud University, Nijmegen, the Netherlands; c Donders Institute for Brain, Cognition and Behaviour, Department of Psychiatry, Radboud university medical center, Nijmegen, the Netherlands; d Depression Expertise Centre, Pro Persona Mental Health Care, Nijmegen, the Netherlands; e Department of Head and Skin, Ghent University, Ghent, Belgium; f Department of Old-age Psychiatry, GGNet Mental Health, Apeldoorn, the Netherlands; g Department of Psychiatry, Radboud university medical center, Nijmegen, the Netherlands; h Depression Association, Utrecht, the Netherlands; i Trimbos Institute, Utrecht, the Netherlands; j SeniorBeter, Nijmegen, the Netherlands

**Keywords:** Cognitive control, Cognitive control training, Late-life depression, Depression, Working memory training

## Abstract

**Background:**

Late-life depression (LLD) is a prevalent and debilitating condition associated with a poor long-term prognosis and high rates of chronicity. Cognitive control impairments are common in LLD and contribute to symptom persistence, yet they are not sufficiently targeted by standard treatments. We investigated whether adding a brief Cognitive Control Training (CCT) program to treatment as usual (TAU) improved outcomes in older adults with LLD.

**Methods:**

In this multicenter, single blind randomized controlled trial, 90 older adults diagnosed with LLD were randomized to eight sessions of CCT (*N* = 49) or an active control training (ACT; *N* = 41) over four weeks, in addition to TAU. Assessments were conducted at baseline, post training (approximately one month after baseline), and at three-, six- and twelve month follow-up. The primary outcome was self-reported depressive symptoms, and secondary outcomes included rumination, emotion regulation strategy use, and cognitive functioning.

**Results:**

A mixed-effects model revealed no significant difference in the reduction of depressive symptoms between the CCT and ACT conditions over the twelve-month follow-up period. Both conditions improved on symptom severity, but no significant differences were found for any of the secondary outcomes.

**Discussion:**

Contrary to our hypothesis, CCT did not yield more clinical improvement in depressive or cognitive outcomes in older adults. Potential explanations include suboptimal intervention dose/intensity, age-related reductions in neural plasticity and processing speed that may limit trainability, and high baseline severity and multimorbidity typical of specialized care.

**Conclusion:**

In this multicenter trial, adding CCT to TAU did not confer clinical benefits for late-life depression. Future research should investigate different dosage strategies and identify patient subgroups most likely to benefit.

## Highlights

• Evaluated online Cognitive Control Training (CCT) for late-life depression.
• 90 adults had 8 sessions of CCT or an active control alongside usual care.
• Both groups had comparable decreases in depressive symptoms over 12 months.
• CCT yielded no significant differences in rumination or cognitive outcomes.
• Brief, single-task CCT lacks clinical efficacy for severe older depression.

## 1 Introduction

Late-life depression (LLD), defined as major depressive disorder (MDD) in older adults aged 60 and over, represents a significant and growing public health concern (Djernes, 2006). The condition is characterized by a poor long-term prognosis, with higher rates of chronicity, relapse, and mortality (Jeuring et al., 2018; Kessler et al., 2009; Mitchell and Subramaniam, 2005). Standard treatments, including pharmacotherapy and psychotherapy, often show diminished efficacy in older adults, with studies reporting recovery rates as low as 18% over two years, compared to around 80% in younger adults (Calati et al., 2012; Comijs et al., 2015; Spijker et al., 2002; Schaakxs et al., 2018). Other established interventions, such as electroconvulsive therapy (ECT) or repetitive transcranial magnetic stimulation (rTMS), also offer effective alternatives for treatment-resistant LLD (Van Rooij et al., 2020; Zhang et al., 2022), but these interventions can be resource-intensive, require clinical visits, and sometimes carry a higher side-effect burden. This treatment gap highlights an urgent need for effective interventions that target mechanisms contributing to the persistence of LLD.

Cognitive deficits are common in LLD, particularly in the domain of cognitive control (i.e., executive functions that enable goal-directed behaviour, such as working memory, attention, and inhibition; Morimoto et al., 2015; Riddle et al., 2017). These deficits are linked to alterations in the brain's cognitive control network and are thought to weaken an individual's ability to regulate negative emotions, contributing to dysfunctional repetitive negative thinking like rumination (Alexopoulos et al., 2012). Importantly, impaired cognitive control in LLD has been associated with a poorer response to antidepressant medication, suggesting cognitive functioning is a crucial target for improving effectiveness of standard treatment (Alexopoulos et al., 2015; Morimoto et al., 2015; Pimontel et al., 2015). Consequently, there is a need for mechanistic augmentation strategies. By adding an intervention specifically designed to target cognitive deficits, we aim to address this residual cognitive vulnerability that standard care often leaves untreated, potentially enhancing the overall clinical response.

One possible way of improving cognitive control is with Cognitive Control Training (CCT), which refers to computerized tasks designed to strengthen cognitive control through repetitive practice (Vander Zwalmen et al., 2022). Recent meta-analyses suggest that CCT yields small to moderate improvements in depressive symptoms and cognitive performance in individuals with depression (Motter et al., 2016; Legemaat et al., 2021). A frequently used operationalization of CCT is the adaptive Paced Auditory Serial Addition Task (aPASAT; Siegle et al., 2007, Siegle et al., 2014). In this training, participants are presented with auditory stimuli (numbers ranging from 1 to 9) and are asked to continuously respond to the sum of the last two heard digits. This requires participants to keep these auditory stimuli in their working memory, perform the addition, and discard previous (i.e., no longer relevant) stimuli, such as previously heard auditory stimuli and previous sums. By increasing the speed of stimulus presentation by 100 ms after every four consecutive correct responses and decreasing the speed by 100 ms following every four consecutive incorrect responses, this adaptive training continuously adjusts difficulty to participant performance, thereby engaging cognitive control. For an overview of prior aPASAT literature in depression, we refer to a recent systematic review and meta-analysis (Vander Zwalmen et al., 2024).

While CCT has shown promise in adult populations, translating these benefits to specifically aid older adults presents unique challenges due to age-related neural changes. Consequently, evidence regarding its efficacy in older adults remains mixed and appears highly dependent on the population's clinical status. For instance, Vanderhasselt et al. (2020) conducted a proof-of-concept trial in healthy older adults and found that while participants improved on the training task itself (i.e., task-specific transfer), this did not translate to improvements in emotion regulation or affect. This suggests that in the absence of cognitive or emotional deficits, CCT may lack a target to remediate. Conversely, in a sample of older adults with MDD, Brunoni et al. (2014) reported that CCT (combined with transcranial direct current stimulation) was associated with significant symptom reduction. Crucially, they found that older patients who demonstrated greater improvement in task performance during training showed the largest clinical benefits. Together, these findings suggest that while healthy aging brains may show limited transfer, depressed older adults might especially benefit from CCT, provided they can successfully engage with the training intervention.

Prior work emphasizes that individual differences could influence responsiveness to CCT (von Bastian et al., 2022). Two competing theoretical frameworks describe this variability: the compensation model suggests that individuals with lower baseline cognitive abilities have the most room for improvement and thus benefit most (Karbach et al., 2017). Meanwhile, the magnification model posits that a certain threshold of cognitive efficiency is required to successfully engage with, and benefit from training, meaning those with higher baseline functioning (i.e., cognitive reserve) will derive the most gain (Borella et al., 2017; Traut et al., 2021). Notably, evidence from working-memory training suggests that individuals with higher baseline cognitive control tend to benefit more, whereas those with very low control often struggle to improve (Basak and O'Connell, 2016). Given that cognitive control declines with age (Forte et al., 2024), this raises the possibility that some older adults may enter training below the threshold needed to fully engage with CCT.

To evaluate the efficacy of CCT, the inclusion of an active control condition (ACT) is essential (Koster et al., 2017). Previous meta-analyses indicate substantial placebo response rates in depression treatments, ranging from 35% to 40% (Furukawa et al., 2016; Enck and Zipfel, 2019). Without an active control, it remains unclear whether observed improvements are attributable to the specific remediation of cognitive control or to non-specific factors such as treatment expectancy, researcher interaction, and behavioral activation (Cuijpers and Cristea, 2015; Nguyen et al., 2019). Therefore, contrasting CCT solely with Treatment As Usual (TAU) would fail to isolate the mechanistic effects of the training task itself. To determine if CCT offers a distinct cognitive benefit beyond the non-specific effects of behavioral activation and expectancy inherent in engaging with a digital intervention, a rigorously matched active control condition is required.

Despite recent trials in adults, no long-term CCT trial using an active control condition has been conducted for an LLD population, even though this group faces pronounced cognitive control deficits and poor treatment outcomes, making them especially in need of validated, mechanistically targeted interventions. Therefore, the primary aim of the present study was to evaluate in a multicenter randomized controlled trial whether adding CCT to TAU would reduce depressive symptoms more effectively, compared to an active control, in adults with LLD. We hypothesized that participants receiving CCT would demonstrate significantly greater depressive symptoms reduction at one, three, six, and twelve months post-training. Since depression can be characterized by difficulties in disengaging from negative thought content (Koster et al., 2011), cognitive control deficits could be closely linked to elevated rumination, reduced emotion regulation, and poorer functional outcomes. Our secondary objectives were to examine the intervention's effects on rumination, cognitive and emotional functioning, and to assess whether baseline characteristics like age or depression severity moderated the treatment effect.

## 2 Method

The methods for this study followed the pre-registered protocol (see Meuleman et al., 2021). The study was a multicenter, single-blind, randomized controlled trial, and participants were recruited from four mental health care centers (Pro Persona (*N* = 33), GGNet (*N* = 36), Senior Beter (*N* = 9) and Radboud university medical center (*N* = 12) in The Netherlands between 7 May 2019 and 19 December 2022. All procedures were approved by the regional Medical Ethical Committee Arnhem-Nijmegen (NL67671.091.180), and all participants provided written informed consent prior to participation.

### 2.1 Participants

Participants were adults aged 60 years or older who were receiving TAU for a current DSM-IV diagnosis of MDD at specialized mental healthcare centers in The Netherlands. Treatment as usual (TAU) was provided in accordance with the Dutch multidisciplinary guidelines for the treatment of LLD in specialized mental healthcare. As detailed in Table 1, approximately half of the participants were taking antidepressant medication, and around 70% were engaged in psychotherapy. The most frequently administered psychotherapy was Cognitive Behavioral Therapy (CBT), followed by Schema Therapy and Interpersonal Psychotherapy (IPT). A substantial subset of participants received treatment within intensive clinical frameworks, including multidisciplinary day treatment programs, intensive group therapy, or inpatient clinical care. Pharmacological treatment primarily consisted of antidepressants (including SSRIs, SNRIs, and TCAs), occasionally augmented with lithium. Supportive psychiatric care (e.g., consultations with specialized psychiatric nurses) was also frequently provided alongside these therapies. Eligible patients were approached by their treating clinicians during both the diagnostic intake and ongoing treatment phases. Following preliminary consent, interested patients were referred to the researchers to receive comprehensive information and provide formal written informed consent.

**Table 1:** Group characteristics.

Characteristic	CCT (N = 49)	ACT (N = 41)
Age (*SD*)	71.5 (*6.6*)	71.6 (*6.8*)
Sex (male:female)	26:23	16:25
Education level		
Primary school	3	1
Lower secondary school	37	30
Upper secondary school	7	8
Higher education	2	2
Age of first depressive episode (*SD*)	24.8 (*11.3*)	26.2 (*12.9*)
Mean number of past depressive episodes (*SD*)	4.2 (*5.1*)	3.2 (*3.4*)
Current ADM use (%)	46.9	56.1
Current psychotherapy (%)	73.5	65.9

*Note*: ACT: Active Control Training; ADM: Antidepressant Medication; CCT: Cognitive Control Training.

Clinical diagnoses were confirmed by trained clinicians using a structured diagnostic interview, either the Mini International Neuropsychiatric Interview (MINI; Sheehan et al., 1998; Van Vliet and De Beurs, 2007) or the Structured Clinical Interview for DSM-IV (SCID-I; First and Gibbon, 2004). Exclusion criteria included any current psychotic disorder, a history of bipolar disorder, a primary diagnosis of substance or alcohol dependence, or acute suicidal risk as assessed by a clinician. Participants were randomly assigned to either the intervention or active control group using a computer-generated allocation sequence, stratified by baseline depression severity (IDS-SR score: <38, 39–48, or > 49).

### 2.2 Intervention

All participants were instructed to complete eight online training sessions at home over approximately four consecutive weeks using a provided tablet computer. During the baseline assessment, a researcher provided a face-to-face demonstration of the tablet and the assigned training task. Participants completed a brief, supervised practice block to ensure they fully understood the task instructions and could comfortably operate the device before commencing their independent home-based sessions. Throughout the training period, participants had access to ongoing technical support. Support could be reached via phone or email four days a week to assist with any operational issues. The interventions were matched in duration (20 min per session) and presentation format. In the CCT condition, participants completed the aPASAT. To evaluate participants' training progress, we analyzed the median interstimulus interval (ISI) across training sessions. The ISI serves as an adaptive performance index, with shorter intervals reflecting improved task proficiency. For each participant, we examined changes in median ISI over time to assess training progression.

In the ACT condition, participants completed a speed-of-response task designed to control for non-specific intervention effects like computer use and engagement. They were presented with the same auditory stream of digits as the CCT group but were instructed only to tap the number on the touchscreen that corresponded to the single last digit they had heard. This task mimics the sensory and motor demands of the CCT but does not place a significant load on working memory (Hoorelbeke et al., 2021). Following the recent typology proposed by Goldberg et al. (2023), this constitutes a Placebo-Active control condition, which controls for non-specific intervention effects, such as digital device engagement, dedicated time, and treatment expectancy, allowing to isolate the specific incremental value of the working memory load.

### 2.3 Outcomes

The primary outcome, depressive symptom severity, was measured using the Dutch version of the Inventory of Depressive Symptomatology – Self Report (IDS-SR; Rush et al., 2000). The IDS-SR consists of 30 items assessing core symptom domains of depression. It was administered at baseline (T0), post-training (T1; 1 month after training), and at three follow-ups scheduled: 3 (T2), 6 (T3), and 12 months (T4) after the intervention, in line with the study protocol (see Meuleman et al., 2021). In the current sample, the internal consistency of the IDS-SR was good (Cronbach's *α* = 0.84). In the statistical analyses, IDS-SR scores were modeled using linear mixed-effects models. Group, time, and their interaction were included as fixed effects, with a random intercept for participant. In addition, baseline IDS-SR was included as a covariate to adjust for initial differences.

Trait rumination was assessed using the Dutch version of the Ruminative Response Scale (RRS; Nolen-Hoeksema and Morrow, 1991). The questionnaire consists of 22 items measuring the tendency to engage in repetitive and passive thinking about one's depressive symptoms and their causes and consequences. The RRS demonstrated excellent internal consistency (Cronbach's *α* = 0.93) and was administered at baseline (T0) and post-training (T1).

State rumination was assessed with the Breathing Focus Task (BFT; Hirsch et al., 2009). Participants were asked to close their eyes and focus on their breathing for 5 min, during which they heard 12 random auditory probes. After each probe, they indicated whether they were focused on their breathing or experiencing an intrusion. If they reported an intrusion, they categorized it as positive, neutral, or negative. The proportion of negative intrusions relative to the total number of probes served as the outcome measure, with higher proportions indicating greater state rumination. The BFT was administered at baseline (T0) and post-training (T1).

Cognitive coping strategies were measured using the Cognitive Emotion Regulation Questionnaire (CERQ; Garnefski et al., 2001). The CERQ consists of nine subscales, which can be combined into two higher-order factors: adaptive strategies (e.g., positive reappraisal, refocus on planning) and maladaptive strategies (e.g., rumination, catastrophizing). The adaptive (Cronbach's *α* = 0.76) and maladaptive (Cronbach's *α* = 0.79) subscales both demonstrated acceptable internal consistency. The CERQ was administered at baseline (T0) and post-training (T1) in accordance with the study protocol.

Cognitive control was assessed with a standardized version of the Paced Auditory Serial Addition Task (PASAT; Gronwall, 1977), where single-digit numbers are presented sequentially, and participants are instructed to continuously add the most recent number to the one immediately preceding it. The standardized PASAT has three blocks of 60 trials, at 3000 ms, 2000 ms and 1500 ms. Performance accuracy was quantified as the proportion of correct responses. The PASAT was administered at baseline (T0) and post-training (T1).

Executive functioning and inhibitory control were assessed using the Random Number Generation (RNG) task (Jahanshahi et al., 2000; Peters et al., 2007). Participants were instructed to generate random sequences of numbers between 1 and 10. To quantify randomness quality, we calculated first-order redundancy, which reflects deviations from an even digit distribution. A value of 0 indicates a perfectly uniform distribution (optimal randomness), whereas higher values indicate greater redundancy and thus lower randomness quality. The RNG was administered at baseline (T0) and post-training (T1).

Participants completed two Stroop tasks to assess the capacity to inhibit attention to interfering information: a Color Stroop and an Emotional Stroop (Williams et al., 1996). In the Color Stroop, participants were asked to name the ink color of color words that were either congruent (e.g., the word red printed in red), incongruent (e.g., the word red printed in blue), or neutral (e.g., non-color strings or “X”s). In the Emotional Stroop, participants named the color of words with positive, negative, or neutral emotional valence. According to the protocol, both reaction times (RTs) and accuracy were to be analyzed. However, due to practical problems during data collection (two different Stroop versions were used across sites and technical issues prevented complete data capture), RTs were not consistently recorded, and a large proportion of data was missing. Consequently, only accuracy interference scores could be analyzed. Error rates (proportion incorrect) were computed for each condition, and interference scores were calculated as the difference between incongruent and congruent trials (Color Stroop), and between negative or positive and neutral trials (Emotional Stroop).

### 2.4 Sample size calculation

We originally aimed to detect a medium effect size (Cohen's *d* = 0.45) on the primary outcome (IDS-SR) with 80% power and an alpha of 0.05 (two-tailed). The calculation accounted for a longitudinal design with one baseline and four follow-up assessments, assuming a correlation of *r* = 0.50 between timepoints. Additionally, we wanted to correct for the nesting of participants within clinical centers (clustering). This resulted in a required sample size of 52 participants per condition (*N* = 104 in total). For more details, we refer to our study protocol (see Meuleman et al., 2021). After consultation with the grant provider, we conducted a recalibrated power analysis, based on observed characteristics in the accrued baseline data when a total sample of *N* = 50 was acquired, replacing several assumed parameters by empirical estimates. Using a mean cluster size of 1.9, a coefficient of variation of 1.05, and an ICC of 0.10, the resulting design effect was 1.32, yielding an adjusted target sample size of approximately 45 participants per group (N ≈ 90).

### 2.5 Statistical analyses

Statistical analyses were performed using R (v4.4.2) and the study followed the intention-to-treat (ITT) principle. The primary outcome (IDS-SR) was analyzed using linear mixed-effects models to account for the longitudinal data structure. Fixed effects included Group, Time, and the Group × Time interaction, while a random intercept for participant was included to account for repeated measures. Baseline IDS-SR scores were included as a covariate to adjust for initial severity. To account for the multicenter design, we initially fitted a three-level model including a random intercept for the study center. However, a likelihood ratio test indicated that inclusion of the center level did not significantly improve model fit (*χ*^2^(1) = 0.14, *p* = .710). Consequently, we report the results from the more parsimonious two-level model without clustering by center. As mixed-effects models were used, no data imputation was conducted. For secondary outcomes assessed at a single follow-up point (RRS, BFT, CERQ, PASAT, RNG, and Stroop), Analysis of Covariance (ANCOVA) was performed on available cases. These models compared post-training scores (T1) between groups, with the respective baseline score (T0) entered as a covariate, allowing testing of adjusted post-intervention group differences while accounting for baseline severity. In line with our protocol, moderation analyses were conducted using linear mixed-effects models to the primary model to assess if age or baseline depression severity influenced the results.

Additionally, an exploratory post-hoc analysis was conducted to examine whether training progress predicted clinical outcomes. We computed individual learning trajectories (slopes of median ISI across sessions). Using k-means clustering, we classified participants based on these trajectories to determine if the rate of improvement on the aPASAT training was associated with depressive symptom reduction. A linear mixed-effects model was then used to compare IDS-SR score trajectories between these two subgroups, including baseline severity as a covariate.

In line with the study protocol, a health-economic evaluation was planned, following CHEERS (Husereau et al., 2013) guidelines. Both cost-effectiveness analysis (CEA) and cost-utility analysis (CUA) were to be conducted from healthcare and societal perspectives, assessing healthcare utilization, out-of-pocket costs, and productivity losses. Incremental cost-effectiveness ratios (ICERs) were to be estimated for IDS-SR response and remission. In addition, a budget impact analysis (BIA) was planned to evaluate the financial consequences of scaling up the intervention to various levels of coverage.

## 3 Results

### 3.1 Group characteristics

**Fig. 1:**
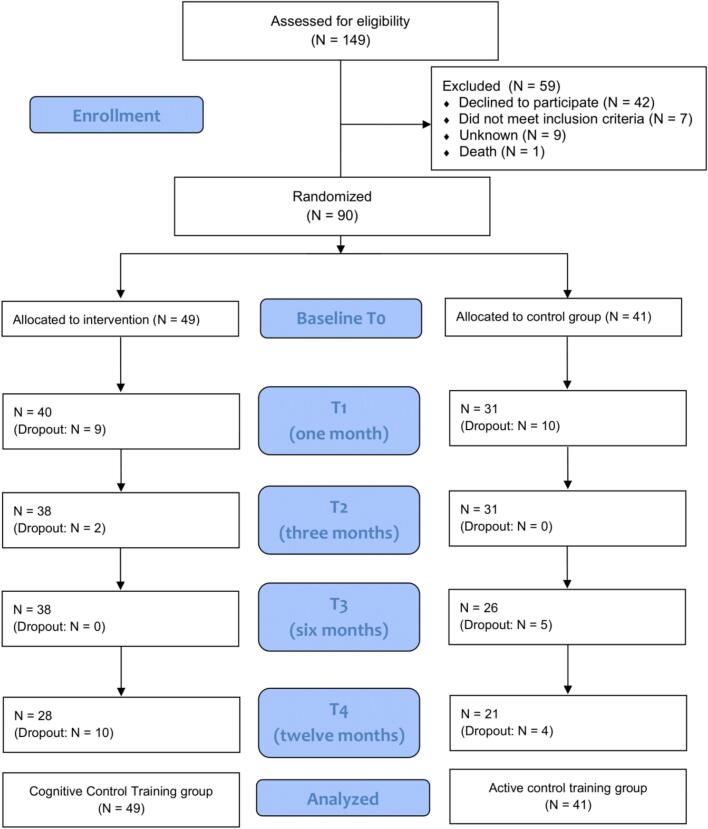
CONSORT flow diagram.

A total of 98 individuals were assessed for eligibility, of whom 90 met the inclusion criteria and were randomized into the CCT group (*N* = 49) or the ACT group (*N* = 41). The flow of participants through each stage of the trial is presented in the CONSORT diagram (see Fig. 1). Baseline demographic and clinical characteristics were well-balanced across the two groups (see Table 1) and no clinically relevant imbalances were observed at baseline. The mean (*SD*) age of the complete sample was 71.6 (*6.7*) years and 53.3% of participants were female. The CCT group on average completed 7.2 training sessions (SD = 1.8), suggesting the training protocol was feasible. In the ACT condition, participants on average completed 7.2 sessions (SD = 2.0). Table 2 provides an overview of the IDS-SR scores, and Table 3 summarizes the secondary self-report questionnaire outcomes and task performance data across groups over time.

**Table 2:** Means of IDS-SR scores per group over time.

IDS-SR scores	CCT	ACT
T0 – Mean (*SD*)	33.27 (*11.95*)	35.59 (*12.95*)
T1 – Mean (*SD*)	29.33 (*13.46*)	29.57 (*14.90*)
T2 – Mean (*SD*)	30.73 (*14.33*)	28.97 (*13.54*)
T3 – Mean (*SD*)	29.32 (*14.75*)	28.19 (*14.38*)
T4 – Mean (*SD*)	28.25 (*13.99*)	28.24 (*16.04*)

*Note*: *IDS-SR*: Inventory of Depressive Symptomatology; T0: baseline; T1: Post-training (approx.. 1 month); T2: 3 months after training; T3: 6 months after training; T4: 12 months after training.

**Table 3:** Means of secondary outcomes per group over time.

Variable	CCT T0	CCT T1	ACT T0	ACT T1	Test Statistic	*p*-value	Adjusted R^2^
RRS (*SD*)	58.02 (*13.85*)	57.63 (*16.73*)	60.11 (*14.94*)	55.39 (*13.58*)	*F*(1, 57) = 0.24	0.620	0.31
BFT (*SD*)	0.29 (*0.36*)	0.28 (*0.32*)	0.51 (*0.35*)	0.32 (*0.37*)	*F*(1, 69) = 0.00	0.995	0.04
CERQ – adaptive (*SD*)	48.68 (*9.61*)	52.46 (*11.46*)	46.18 (*12.10*)	49.73 (*10.52*)	*t*(60) = −0.58	0.568	0.55
CERQ – maladaptive (*SD*)	38.39 (*10.21*)	38.63 (*12.06*)	37.38 (*7.98*)	39.23 (*10.04*)	*t*(60) = 0.91	0.367	0.63
PASAT (*SD*)	14.06 (*8.81*)	42.99 (*19.93*)	11.36 (*10.87*)	15.76 (*11.96*)	*F*(1, 62) = 40.18	< 0.001	0.44
RNG (*SD*)	2.10 (*1.58*)	2.05 (*1.41*)	2.49 (*2.08*)	2.05 (*1.71*)	*F*(1, 67) = 0.04	0.841	0.10
Color Stroop – Interference ACC	−5.21 (*13.94*)	−2.74 (*5.02*)	−0.05 (*0.07*)	−0.03 (*0.06*)	*F*(1, 62) = 0.02	0.895	0.01
Emotional Stroop – Interference ACC negative	−0.09 (*1.13*)	0.29 (*0.88*)	0.00 (*0.02*)	0.00 (*0.01*)	*F*(1, 61) = 0.83	0.366	0.02
Emotional Stroop – Interference ACC positive	0.27 (*1.29*)	−0.06 (*1.66*)	0.00 (*0.02*)	0.00 (*0.01*)	*F*(1, 59) = 0.07	0.790	0.02
MOCA	26.02 (*2.50*)	/	25.03 (*2.98*)	/	/	/	/

*Note*: *ACC*: Accuracy in %; *ACT*: Active Control Training; *BFT*: Breathing Focus Task; *CCT*: Cognitive Control Training; *CERQ*: Cognitive Emotion Regulation Questionnaire; *IDS-SR*: Inventory of Depressive Symptomatology; *MOCA*: Montreal Cognitive Assessment; *PASAT* Paced Auditory Serial Addition Task; *RNG*: Random Number Generation task; *RRS*: Ruminative Response Scale*; RT*: Reaction Time in milliseconds; *STROOP*: Stroop task; T0: baseline; T1: Post-training (approx.. 1 month).

### 3.2 Primary outcome

Across timepoints, IDS-SR scores decreased in both groups. The baseline-adjusted mixed-effects model revealed a significant main effect of Time (*χ*^2^(4) = 19.44, *p* < .001). Relative to baseline, scores were significantly lower at post-training (*b* = −5.39, SE = 1.76, *t*(278.31) = −3.06, *p* = .002), 3-month follow-up (*b* = −6.04, SE = 1.74, *t*(277.98) = −3.46, *p* < .001), 6-month follow-up (*b* = −6.97, SE = 1.85, *t*(281.08) = −3.77, *p* < .001), and 12-month follow-up (*b* = −4.51, SE = 1.99, *t*(282.54) = −2.26, *p* = .025).

The model also showed a significant effect of baseline scores (*b* = 0.85, SE = 0.05, *t*(88.46) = 16.40, *p* < .001), indicating that higher initial severity predicted higher scores across follow-ups. No significant main effect of Group emerged (*b* = −0.35, SE = 1.82, *t*(262.86) = −0.19, *p* = .850), and the Group × Time interaction was not significant at any timepoint (*χ*^2^(4) = 4.54, *p* = .338; all individual timepoint *p*'s > 0.09). Thus, both groups showed comparable decreases in IDS-SR over time.

Attrition by the 12-month follow-up was notable, with 28 of 49 participants remaining in the CCT condition and 21 of 41 in the ACT condition. An attrition analysis revealed that participants who dropped out before the 12-month timepoint had significantly higher baseline depressive severity (IDS-SR *M* = 37.39) compared to those who completed the final follow-up (IDS-SR *M* = 31.76), *t*(87.47) = −2.24, *p* = .027. This indicates that more severe baseline depression was associated with a higher likelihood of dropout. Importantly, the linear mixed-effects models used for the primary analyses inherently account for this attrition by utilizing Maximum Likelihood Estimation and explicitly including baseline IDS-SR scores as a covariate.

**Fig. 2:**
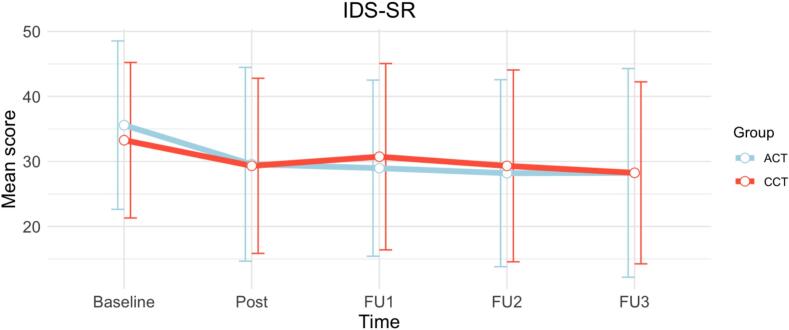
Means of IDS-SR scores over time for both groups. *Note*: Error bars represent standard deviations. *Post*: one month after baseline, *FU1*: follow-up 1, three months after baseline, *FU2*: follow-up 2, six months after baseline, *FU3*: follow-up 3, twelve months after baseline. *ACT*: Active control training, *CCT*: Cognitive Control Training, *IDS*-*SR*: Inventory of Depressive Symptomatology – Self Report.

To test for an overarching linear trend of symptom decline across the 12-month period, we also modeled time as a continuous variable. This continuous model confirmed the findings of the categorical approach: there was evidence of a significant linear decline in IDS-SR scores across assessments (*b* = −1.34, SE = 0.44, *t*(291.32) = −3.03, *p* = .003), independent of group. Across both analytical approaches, baseline severity consistently predicted higher follow-up scores, and the Group × Time interaction remained non-significant (*p* = .334). Model fit indices suggested that the baseline-adjusted model explained substantial variance (conditional R^2^ = 0.74). Exploratory moderation analyses were conducted to determine if the treatment effect was influenced by baseline severity or age. No significant three-way interactions were found (both *p*'s > 0.05), suggesting that neither initial symptom severity nor age moderated the clinical response to CCT. For a visualization of the IDS-SR scores over time, see Fig. 2.

### 3.3 Secondary outcomes

An ANCOVA predicting follow-up RRS scores from condition, controlling for baseline scores, showed that baseline RRS strongly predicted follow-up (*b* = 0.58, *SE* = 0.11, *t*(57) = 5.28, *p* < .001). There was no significant main effect of condition (*F*(1, 57) = 0.24, *p* = .62), indicating that participants in the CCT and ACT conditions did not differ on post-intervention RRS scores when controlling for baseline rumination. The model explained 31% of the variance in follow-up RRS scores (adjusted R^2^).

For the BFT, baseline scores significantly predicted post-training proportions of negative intrusions (*b* = 0.24, SE = 0.11, *t*(69) = 2.16, *p* = .034). The main effect of condition was non-significant (*F*(1, 69) = 0.001, *p* = .995), indicating no differences between the two conditions at post-training (*b* = 0.00, SE = 0.08, *t*(69) = 0.01). The overall model explained little variance (adjusted R^2^ = 0.04).

When running the ANCOVA models for the CERQ, both baseline adaptive and maladaptive strategy use predicted post-training scores, with higher baseline scores predicting higher post-intervention scores (adaptive: *b* = 0.75, *SE* = 0.09, *t*(60) = 8.71, *p* < .001; maladaptive: *b* = 0.65, *SE* = 0.06, *t*(60) = 10.36, *p* < .001). The main effects of conditions were also not significant for both the adaptive (*b* = −1.11, *SE* = 1.93, *t*(60) = −0.58, *p* = .568, $R_{\mathrm{Adj}}^{2}$ = 0.55) and maladaptive (*b* = 1.28, *SE* = 1.40, *t*(60) = 0.91, *p* = .367, $R_{\mathrm{Adj}}^{2}$ = 0.63) subscales.

Baseline PASAT scores significantly predicted post-training accuracy, and higher baseline accuracy was associated with higher post-training accuracy (*b* = 0.54, SE = 0.20, *t*(62) = 2.70, *p* = .009). The ANCOVA indicated a significant main effect of condition, *F*(1, 62) = 40.18, *p* < .001. As expected, participants in the CCT condition showed significantly higher post-training accuracy compared to the ACT condition, when controlling for baseline performance (*b* = 0.26, SE = 0.04, *t*(62) = 6.34, *p* < .001). The overall model accounted for 44% of the variance in post-training PASAT scores.

Higher baseline redundancy on the RNG was associated with higher post-training redundancy (*b* = 0.29, SE = 0.09, *t*(67) = 3.17, *p* = .002). There was no significant main effect of group, *F*(1, 67) = 0.04, *p* = .841, indicating that the intervention and control conditions did not differ in post-training redundancy. The overall model accounted for only 10% of the variance in post-training redundancy (adjusted R^2^ = 0.10).

Due to substantial missing data (up to 91%), Stroop models using RTs could not be estimated. Accuracy interference scores were analyzed, but no significant condition effects emerged. On the Color Stroop, after adjusting for baseline, there was no condition effect on accuracy interference at post-test (*F*(1, 62) = 0.02, *p* = .895, $R_{\mathrm{Adj}}^{2}$ = 0.01). Similarly, on the emotional Stroop, no condition effects were found for negative interference (*F*(1, 61) = 0.83, *p* = .366, $R_{\mathrm{Adj}}^{2}$ = 0.02), or positive interference (*F*(1, 59) = 0.07, *p* = .790, $R_{\mathrm{Adj}}^{2}$ = 0.02). Across all analyzable Stroop outcomes, results provided no evidence for differential training effects.

### 3.4 Training progress

**Fig. 3:**
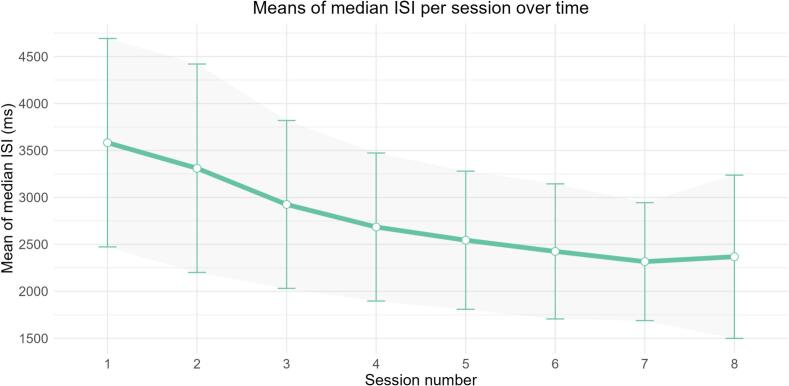
Means of aPASAT median interstimulus interval per CCT session. *Note*: Error bars represent standard deviations. The ACT control condition is not included in this plot as these participants performed no aPASAT training.

In line with prior aPASAT research, participants showed an initial gradual reduction in median ISI, indicating improved task performance over time (Vander Zwalmen et al., 2025). Fig. 3 visualizes the means of the median ISI per session over time for the CCT condition. The exploratory k-means clustering analysis on the slope values yielded division into two subgroups: rapid improvers (characterized by steep negative slopes indicating fast learning) and slow/non-improvers (characterized by flatter slopes indicating limited training improvement). A linear mixed model modeling IDS-SR scores in function of time and improver group, including baseline depressive scores as covariate, found no overall differences between improvers and non-improvers (*p* = .063) and no time × improver group interaction (*p* = .102), suggesting that stronger training gains were not associated with more clinical improvement.

### 3.5 Health-economic evaluation

Although the trial protocol specified a full health-economic evaluation, this was not performed. As the trial did not demonstrate any clinical advantage of CCT over sham training on primary or secondary outcomes, there was no incremental effectiveness to relate to costs. Accordingly, cost-effectiveness, cost-utility, and budget impact analyses were deemed not informative and were not undertaken.

## 4 Discussion

This multicenter single blind randomized controlled trial was designed to evaluate the efficacy of an adaptive CCT program as an add-on treatment for LLD. Contrary to our primary hypothesis, we found no evidence that adding four weeks of CCT to TAU was superior to an active control condition with TAU in reducing depressive symptoms over a 12-month follow-up period. This null result was consistent across all secondary clinical, cognitive, and functional outcomes. These findings suggest that the current CCT protocol, consisting of a brief, home-based, single-task training delivered as an add-on to standard care, may not be sufficient for older adults. However, the absence of effects does not preclude the potential of cognitive interventions for LLD more broadly. Rather it suggests that future studies may need to consider adjusting key elements of the protocol, such as training intensity, task composition, delivery format, or patient selection criteria We discuss these considerations in the following sections. Our results stand in contrast to earlier studies of the aPASAT that suggested clinical benefits in MDD (Siegle et al., 2014), but align more closely with CCT literature regarding older adults specifically. For instance, a meta-analysis by Lampit et al. (2014) on computerized cognitive training in healthy older adults found small and significant effect sizes on working memory and processing speed, but no significant effects for executive functions or attention.

### 4.1 Sample characteristics

A critical factor in interpreting these findings is the clinical severity of LDD in the sample. Participants were recruited from specialized mental healthcare centers, implying a population with moderate-to-severe, complex, or treatment-resistant depression, which is also supported by the high baseline depression scores. Nearly half were on antidepressant medication and the majority were receiving psychotherapy. In such a sample, the effects of TAU may limit the detectable added value of a brief computerized add-on intervention. While our sample size precludes adequately powered empirical tests of subgroup differences, we hypothesize that intervention effects might vary based on baseline clinical severity. It remains an open question for future, larger-scale research whether CCT might yield stronger clinical benefits in less severe, primary-care populations, or conversely, if highly specific subgroups of treatment-resistant patients might uniquely benefit from extended dosing. Furthermore, demonstrating the efficacy of add-on interventions in severe depression remains a challenge: the incremental benefit of CCT may be small relative to the effects of pharmacological and psychotherapeutic interventions. Consequently, a ‘signal-to-noise’ issue arises, where the specific effects of CCT are overshadowed by the broader treatment regimen and the chronicity of the disorder. Additionally, the limited improvement in depression scores over time supports the notion that our sample represented a largely treatment-resistant group.

### 4.2 Intervention-related factors

Furthermore, the intervention characteristics, specifically dosage and task specificity, may have been insufficient for this population. Eight sessions of a single-domain working memory task may not be a potent enough training to induce (lasting) neuroplastic changes. It could be the case that, for cognitive training to be effective, it needs to target multiple cognitive domains simultaneously to foster better generalization (Kueider et al., 2012; Nguyen et al., 2019). However, using multi-domain paradigms come with a methodological trade-off and can make it difficult to disentangle which specific task components drive clinical effects. Additionally, training performance should not necessarily be interpreted as an indicator of cognitive control gains. In older adults, performance plateaus on the aPASAT may reflect age-related limits in processing speed and motor response time rather than a maximization of working memory capacity (Tombaugh, 2006). Therefore, eight sessions may have been sufficient to reach a psychomotor limit, but insufficient to consolidate the treatment gains required for far-transfer to other cognitive and/or emotional outcomes. Moreover, it could also be the case that the 20-min sessions were not an optimal training duration, and that more frequent (but potentially shorter) sessions could lead to different results. Notably, our results contrast with previous findings in LLD where training improvement predicted clinical response (Brunoni et al., 2014). In the current study, even ‘rapid improvers’ did not show greater symptom reduction. This dissociation between task improvement and clinical gain suggests that observed performance gains may reflect task-specific strategy learning rather than a genuine restoration of the cognitive control network required for emotion regulation (Dunning and Holmes, 2014; Laine et al., 2018). This highlights the challenge of bridging the gap between near-transfer (i.e., task improvement) and far-transfer (i.e., generalization to clinical symptoms) in cognitive training interventions (von Bastian et al., 2022).

The setting of the intervention also warrants consideration. A notable strength of this study was the home-based administration of the training, which enhances ecological validity and potential for transfer effects. However, the digital nature of the intervention presented challenges. Moderator analyses in healthy older adults have previously revealed that home-based administration was less effective compared to group-based training (Lampit et al., 2014). Indeed, unguided, online interventions often result in diminished or absent effects, potentially due to reduced treatment adherence (Akdemir et al., 2024). While technical support was provided, the lack of face-to-face therapeutic coaching may have reduced engagement compared to a clinical setting. Methodological Considerations.

A further explanation for the observed null effects may lie in the overestimation of the expected treatment effect. The original power calculation was based on earlier randomized trials reporting medium-to-large effects of CCT on depressive symptoms. However, a recent meta-analysis of aPASAT-based CCT trials reported substantially smaller pooled effect sizes on depressive symptoms (Vander Zwalmen et al., 2024), indicating that the treatment effect may be smaller than previously anticipated. If the actual effect of adjunctive CCT in LLD is indeed in the small range, the present trial, despite careful design and recalibrated power estimates, was likely underpowered to reliably detect such effects.

While not directly assessed in the current study, biological aging processes may also provide a hypothetical explanation for the limited impact of the intervention. As cognitive control tends to peak in young adulthood and declines with age, older adults exhibit slower processing and greater interference in cognitive tasks, with inhibitory abilities deteriorating especially in late life (Forte et al., 2024). The lack of transfer to clinical symptoms may be explained by the magnification or reserve model of plasticity (Borella et al., 2017; Karbach et al., 2017; Traut et al., 2021). Unlike younger adults, who may possess the cognitive reserve to leverage training gains, it is possible that in this sample of older adults with LLD, the neural substrates required for cognitive control were insufficiently available to induce the necessary neuroplastic changes.

Finally, the heterogeneity of LLD is a crucial consideration. LLD is increasingly understood as a syndrome with multiple underlying etiologies, including vascular, inflammatory, and neurodegenerative pathways (Alexopoulos, 2019; Morimoto et al., 2015). It is conceivable that CCT is only effective for a specific neurocognitive subtype, such as patients whose depression is primarily driven by “top-down” executive dysfunction (Alexopoulos et al., 2012). In a heterogeneous sample, especially when patients are already receiving multi-modal standard treatments, such a subgroup effect could be undetectable. Future research should aim to stratify patients based on cognitive biotypes to identify those with specific cognitive control deficits who could be more likely to respond to CCT.

### 4.3 Strengths and limitations

The primary strengths of this trial are its robust design, multicenter recruitment enhancing generalizability, a long-term 12-month follow-up, and the inclusion of a well-matched active control group. However, while we excluded severe cognitive impairment, the high likelihood of somatic and psychiatric comorbidity typical of specialized care settings may have reduced the capacity for cognitive change. Additionally, as noted, the digital nature of the intervention may have impacted adherence and engagement compared to face-to-face settings. Furthermore, to limit participant burden, secondary cognitive and affective mechanisms were only assessed immediately post-training, precluding our ability to analyze the long-term trajectories or delayed emergence of near- and far-transfer effects. Moreover, the operationalization of CCT through a single-task paradigm (the aPASAT) constituted a methodological trade-off: while it allowed for mechanistic isolation, it may have lacked the breadth required to induce network-wide clinical changes. A further limitation of the current study is the exclusive reliance on a self-report measure as the primary clinical outcome. While self-reported measures are vital for capturing the subjective burden of depression, evaluating clinical, older populations would also benefit from the inclusion of complementary clinician-rated outcomes to provide a more comprehensive assessment. Lastly, the trial was likely underpowered to reliably detect the smaller effect sizes more recently associated with aPASAT training.

### 4.4 Conclusion and future directions

In conclusion, this RCT found no evidence to support the effectiveness of a CCT program as an add-on treatment for older adults with depression. Given the likelihood that effect sizes for CCT as an add-on intervention are potentially smaller than anticipated, the study may have lacked sufficient statistical power. Future research in cognitive interventions for LLD could explore the use of multiple task paradigms to explore multi-domain training, investigate different intervention dosages and intensities, and focus on identifying patient subgroups who may be most likely to respond to this type of intervention.

## CRediT authorship contribution statement

Conceptualization: Eni S. Becker, Janna N. Vrijsen, Marie-Anne Vanderhasselt, Jan Spijker, Bart Meuleman.

Methodology: Eni S. Becker, Janna N. Vrijsen, Yannick Vander Zwalmen, Marie-Anne Vanderhasselt.

Investigation: Bart Meuleman, Dorine van Driel, Peter Oostelbos.

Formal Analysis: Yannick Vander Zwalmen.

Writing – Original Draft Preparation: Yannick Vander Zwalmen.

Writing – Review & Editing: Yannick Vander Zwalmen, Eni S. Becker, Janna N. Vrijsen, Marie-Anne Vanderhasselt, Jan Spijker, Bart Meuleman, Dorine van Driel, Peter Oostelbos, Paul Naarding, Marleen van Beek, Rose M. Collard, Linda Bolier.

Supervision: Eni S. Becker, Jan Spijker, Marie-Anne Vanderhasselt, Janna N. Vrijsen.

Project Administration: Eni S. Becker, Jan Spijker.

Funding Acquisition: Eni S. Becker, Marie-Anne Vanderhasselt.

## Funding

This project (with project number 852001905 and main applicant Eni S. Becker) is financed by 10.13039/501100001826The Netherlands Organization for Health Research and Development (ZonMW). The funder reviewed the study protocol as part of the application process, but had no role in study design, data collection, analysis or publication. Yannick Vander Zwalmen is supported by a concerted project (GOA) from the Special Research Fund (BOF) of Ghent University (reference number: BOF23-GOA-006).

## Declaration of competing interest

The authors declare that they have no known competing financial interests or personal relationships that could have appeared to influence the work reported in this paper.

## Data Availability

The R code required to run these analyses can be found on OSF: https://osf.io/s7pkr. The data is available upon reasonable request.

## Appendix A Supplementary data

Table A1 Summary of software packages and version numbers.
