# Supplementary material for: Cognitive control training as an add-on treatment for late-life depression: A multicenter randomized controlled trial
Source: Internet Interv. 2026 May 16;44:100951. doi: 10.1016/j.invent.2026.100951 (PMC13197806; doi:10.1016/j.invent.2026.100951)
Supplement: Table A1 — Summary of software packages and version numbers. [file mmc1.docx]

# Supplemental Material

| **Name of software package** | **Version number** |
| --- | --- |
| R | 4.4.2 |
| openxlsx | 4.2.8 |
| lme4 | 1.1-36 |
| emmeans | 1.10.7 |
| lmerTest | 3.1-3 |
| tidyverse | 2.0.0 |
| patchwork | 1.3.0 |
| reshape2 | 1.4.4 |
| MuMIn | 1.48.11 |
| ppcor | 1.1 |
| gtsummary | 2.1.0 |
| car | 3.1-3 |

**Table A1** Summary of software packages and version numbers
